# Emerging roles of ferroptosis in cardiovascular diseases

**DOI:** 10.1038/s41420-022-01183-2

**Published:** 2022-09-20

**Authors:** Kai Wang, Xin-Zhe Chen, Yun-Hong Wang, Xue-Li Cheng, Yan Zhao, Lu-Yu Zhou, Kun Wang

**Affiliations:** 1 Institute for Translational Medicine, The Affiliated Hospital of Qingdao University, College of Medicine, Qingdao University, Qingdao, 266073 Shandong China; 2 State Key Laboratory of Cardiovascular Disease, Heart Failure center, Fuwai Hospital, National Center for Cardiovascular Diseases, Chinese Academy of Medical Sciences, Peking Union Medical College, Beijing, 100037 China; 3 Key Laboratory of Birth Regulation and Control Technology of National Health Commission of China, Shandong Provincial Maternal and Child Health Care Hospital affiliated to Qingdao University, Jinan, 250014 Shandong China

**Keywords:** Cell death, Cardiovascular diseases

## Abstract

The mechanism of cardiovascular diseases (CVDs) is complex and threatens human health. Cardiomyocyte death is an important participant in the pathophysiological basis of CVDs. Ferroptosis is a new type of iron-dependent programmed cell death caused by excessive accumulation of iron-dependent lipid peroxides and reactive oxygen species (ROS) and abnormal iron metabolism. Ferroptosis differs from other known cell death pathways, such as apoptosis, necrosis, necroptosis, autophagy and pyroptosis. Several compounds have been shown to induce or inhibit ferroptosis by regulating related key factors or signalling pathways. Recent studies have confirmed that ferroptosis is associated with the development of diverse CVDs and may be a potential therapeutic drug target for CVDs. In this review, we summarize the characteristics and related mechanisms of ferroptosis and focus on its role in CVDs, with the goal of inspiring novel treatment strategies.

## Facts

- Ferroptosis is a new type of iron-dependent programmed cell death.
- The biological characteristics of ferroptosis include abnormal lipid peroxidation and ROS production.
- Iron metabolism disorder is an important factor in inducing ferroptosis.

## Open Questions

- What are the characteristics of different forms of cell death?
- What mechanism is responsible for the production of ferroptosis?
- Are there any inducers or inhibitors that can target ferroptosis?
- How does ferroptosis participate in diverse cardiovascular diseases?

## Introduction

As the power source of blood flow, the heart can transport blood to all parts of the body and provide oxygen and nutrition to other organs and tissues. It is one of the most important organs in the human body. However, with an unhealthy diet structure and lifestyle, as well as the aggravation of ageing, the incidence and mortality of CVDs are increasing each year, especially in developing countries, and CVDs have become the number one killer [1]. CVDs mainly include myocardial infarction (MI), reperfusion injury, atherosclerosis (AS), hypertension, myocardial hypertrophy, heart failure (HF), diabetic cardiomyopathy (DCM) and doxorubicin (DOX)-induced cardiomyopathy (DIC) [2]. Cardiomyocytes make up the largest proportion of mammalian heart tissue, accounting for three-quarters of the total volume of the heart. The state of cardiomyocytes also affects individual heart function to a certain extent. It is worth noting that, in adult mammals, the proliferation ability of cardiomyocytes in vivo becomes limited, and external adverse factors will dominate the fate of cardiomyocytes. Cell death is a stress response to stimulation by external damage factors. Cardiomyocyte death is involved in regulating cardiac development, senescence and homeostasis, which has important physiological significance [3]. Among them, the common forms of cell death mainly include apoptosis, necrosis, necroptosis, autophagy, pyroptosis and ferroptosis, which were discovered in recent years. A sophisticated regulatory network controls most myocardial cell death [4]. Apoptosis is mainly characterized by cell atrophy, an increase in cytoplasmic density, the disappearance of mitochondrial membrane potential (MMP) and a change in permeability [5], leading to a complete apoptotic body. Necrosis is usually an unexpected and unregulated form of cell death after physical or chemical damage [6]. Necroptosis is also regulated by specific signalling networks. The death receptor TNFR1 plays a key role in the process of necroptosis [7]. Autophagy is a prosurvival mechanism that transfers unwanted or damaged cellular components to lysosomes for degradation and plays an important role in maintaining intracellular metabolic homeostasis [8]. Pyroptosis is considered to be an inflammatory and regulated form of cell death that usually occurs in the defence against exogenous pathogens, such as viruses, bacteria and fungi [9].

The human body contains iron as one of the important elements. Iron in the body exists as haemoglobin (approximately 72%), myoglobin (3%), and other compounds (0.2%), and the rest is reserve iron (25%), which is stored as ferritin in the liver, spleen and bone marrow [10]. Iron is involved in metabolic processes and a variety of life activities, including oxygen transport, cell respiration and electron transfer, DNA synthesis, and immune regulation [11]. The abnormal metabolism of iron leads to disorders of many physiological functions. Ferroptosis was first proposed by Brent R. Stockwell et al., and it was considered an iron-dependent form of cell death [12]. Ferroptosis is characterized by the accumulation of lipid peroxides to lethal levels, resulting in oxidative damage to cell membranes [13]. Ferroptosis is distinct from other forms of cell death in terms of morphology and mechanism (Table 1). An increasing number of studies have reported that ferroptosis plays an important role in CVDs [14, 15]. In this review, we introduce the mechanism of ferroptosis and focus on the research progress of ferroptosis in CVDs to provide ideas for novel treatment strategies.

**Table 1:** Differences in diverse types of cell death.

Cell death forms	Morphological characteristics	Biological characteristics	Immunological effects	Key factors	Classification
Apoptosis	Plasma membrane blistering; Chromatin shrinkage and nuclear fragmentation; Apoptosis bodies	DNA fragments; Activation of the caspase family; Phosphatidylserine exposure	Anti-inflammatory	Caspase family; BCL2 family; Cytc; p53	Programmed cell death
Necrosis	Cell swelling; Plasma membrane rupture; Cell content release	ATP depleted; mPTP on	Promote inflammation	Unknown	Accidental cell death
Necroptosis	Cell swelling; Plasma membrane rupture; Cell content release; Moderate chromatin condensation	ATP decreased; Activated levels of RIP1, RIP3 and MLKL phosphorylation; ROS production	Promote inflammation	RIPK1; RIPK3; MLKL	Programmed cell death
Autophagy	Accumulation of double-membraned autolysosomes	p62 degradation; LC3-I to LC3-II conversion; Enhanced autophagic flux and lysosomal activity	Anti-inflammatory	AMPK; mTOR; ATG5; ATG7; Beclin 1	Programmed cell death
Pyroptosis	Cell swelling (less than necrosis); Pyrosomes form before rupture of the plasma membrane; Cell content release; Mitochondrial integrity unaffected	Activated caspase-1 and GSDMDN; GSDMDN induces pore formation and IL-1β release	Promote inflammation	Caspase1; Caspase11; GSDMD	Programmed cell death
Ferroptosis	Mitochondria are small, and the mitochondrial cristae shrink or disappear, accompanied by rupture of the outer membrane and electron density of the mitochondria. The structure of the plasma membrane and nucleus is intact, and the morphological structure of chromatin remains unchanged.	GPX4 and xCT are inhibited; Deletion of GSH; Iron-dependent lipid peroxidation; ROS production	Promote inflammation	GPX4; Nrf2; TFRC; ACSL4; SLC7A11; NCOA4;Hmox1; p53	Programmed cell death

## Overview of ferroptosis mechanisms

Brent R. Stockwell et al. previously proposed the term ferroptosis in 2012 [12]. They found that erastin induces cell death in Rasv12 cells, an unknown form of cell death that is distinct from apoptosis. Experiments confirmed that Ras-selective lethal (RSL) compound could also induce the cell death phenotype, and the use of apoptosis, necroptosis, autophagy, and pyroptosis inhibitors could not improve the cell death induced by RSL. However, an iron-chelating agent could inhibit this process. This novel form of cell death is therefore considered iron-dependent [16].

**Fig. 1:**
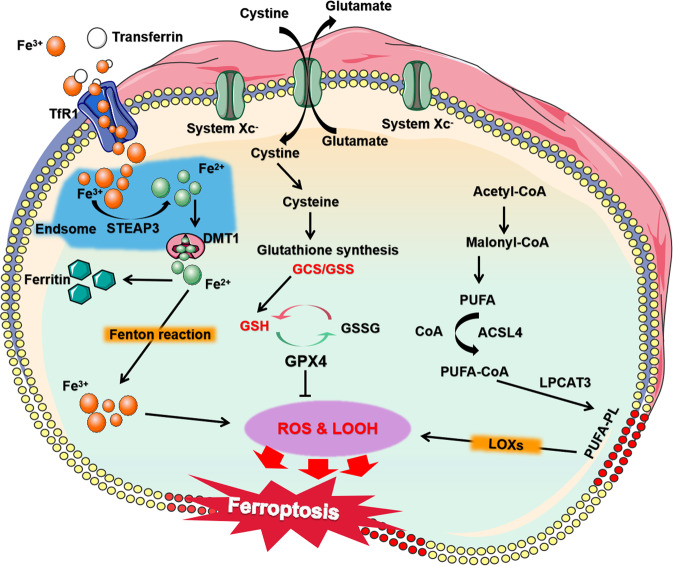
The mechanisms of ferroptosis. Ferroptosis is caused by excessive accumulation of lipid peroxide, ROS production and abnormal iron metabolism. Excessive Fe^2+^ accumulation will cause ROS production and lipid peroxidation. System Xc^−^ can control the transport of cystine to cells and affect GPX4 activity. GPX4 is a key molecule for endogenous inhibition of lipid peroxidation. The arrows indicate promotion, and the blunt-ended lines indicate inhibition. STEAP3 six-transmembrane epithelial antigen of prostate 3, DMT1 divalent metal transporter 1, TfR1 transferrin receptor 1, GSSG glutathione disulfide, ROS reactive oxygen species, LOOH lipid peroxides.

Iron, as an important cofactor in the metabolic process of many enzymes and catalysts for REDOX cycle reactions, participates in diverse key physiological and biochemical processes in vivo [11]. Different physiological conditions and pathological stress may lead to ferroptosis. Among them, abnormal iron metabolism and lipid peroxidation are important factors to induce ferroptosis, and the active state of System Xc^−^ and Glutathione peroxidase 4 (GPX4) is the key mechanism of regulating ferroptosis. Here, we summarize and elaborate on the regulatory mechanism of ferroptosis (Fig. 1).

## Iron metabolism

Iron, a basic element in vivo, is indispensable for life activities as it is involved in the synthesis of many important proteins and enzymes [17]. Intracellular iron overload caused by abnormal iron metabolism is one of the important steps of ferroptosis [18]. In in vivo circulation, iron is mainly in the form of ferric ions (Fe^3+^). Fe^3+^ binds to transferrin and is specifically recognized and transported intracellularly by membrane transferrin receptor 1 (TfR1). Under the action of the six-transmembrane epithelial antigen of prostate 3 (STEAP3), it is reduced to ferrous ion (Fe^2+^), and with the help of divalent metal transporter 1 (DMT1), Fe^2+^ is then released into the cytoplasmic unstable iron pool [19]. In addition to storing Fe^2+^, the iron pool can also store ferric proteins induced by REDOX reactions, such as heme. Ferroportin mediates intracellular iron output to maintain the dynamic balance of iron, and excess iron will remain intracellular as ferritin.

Ferritin usually exhibits non-REDOX activity to prevent cell damage caused by iron overload. However, excessive iron can lead to the accumulation of ROS and induce ferroportin in cells through Fenton and Haber-Weiss reactions [20, 21]. Erastin treatment of H-RasV12 mutant fibrosarcoma cells induces upregulation of TfR1, resulting in increased iron intake. Intracellular ferritin heavy-chain 1 (Fth1) and ferritin light-chain 1 (Ftl1) downregulation also leads to iron overload. Low expression of nuclear receptor coactivator 4 (NCOA4) or autophagy-related (ATG) genes inhibits ferritin degradation and reduces free iron levels, thus limiting oxidative damage caused by ferroptosis [22, 23]. Nuclear factor erythroid 2-related factor 2 (Nrf2) is an important transcription factor that regulates the cellular oxidative stress response and is also a central regulator of maintaining intracellular redox homeostasis [24]. When Nrf2 is activated, the storage of iron increases and inhibits oxidative stress, which blocks ferroptosis [4]. In the mouse cardiomyopathy model induced by DOX, the expression of haem oxygenase-1 (Hmox1) was upregulated, which sped up the degradation of haem and the release of free iron, resulting in ferroptosis and myocardial injury [25]. When the transferrins are exhausted, the metal transporter Slc39a14 can act as a nontransferrin bound iron (NTBI) transporter to introduce iron into cells and induce ferroptosis and tissue fibrosis [26]. Sun et al. found that heat shock protein β-1 (HSPb1) plays an important role in iron metabolism [27]. Protein kinase C can mediate the phosphorylation of HSPb1 and reduce iron-mediated ROS production to resist ferroptosis.

## Lipid peroxidation

Lipid peroxidation is an important marker of ferroptosis. Excessive production of lipid peroxides can lead to loss of stability of the lipid bilayer and disintegration of the cell membrane. The degree of the unsaturated lipid bilayer affects the sensitivity of cells to ferroptosis [28]. Among them, polyunsaturated fatty acids (PUFAs) are most susceptible to peroxidation. The location and content of PUFAs determine the severity of ferroptosis by affecting the degree of intracellular lipid peroxidation. PUFA is attached to the sn-2 site of phospholipids by acyl-coenzyme A (acyl-CoA)-mediated esterification. Acyl-CoA synthase long-chain family member 4 (ACSL4) catalyses the binding of long-chain-PUFA (LC-PUFA) and adrenergic acid to CoA to form PUFA-CoA, which facilitates the entry of LC-PUFA into lipids and membranes [29]. Then, it is esterified into anionic membrane phospholipids by lysophosphatidylcholine acyltransferase 3 (LPCAT3), which changes the remodelling of membrane phospholipids and affects cell ferroptosis [30]. Tammo et al. found that inhibition of ACSL4 could reduce phospholipid-PUFA and inhibit ferroptosis induced by RSL3 [31]. In addition, PUFAs are easily attacked by lipoxygenase (LOX) in chemical structures, resulting in lipid peroxidation and ROS production. Therefore, inhibiting LOX activity may help us treat the damage caused by ferroptosis.

## System Xc^−^

System Xc^−^, a cysteine/glutamate reverse transporter on the cell membrane, is mainly composed of substrate-specific subunit SLC7A11 and regulatory subunit SLC3A2. It has been found that System Xc^−^ is an upstream target regulating ferroptosis [32]. System Xc^−^ can exchange extracellular cystine with intracellular glutamate (ratio 1:1). Then, cystine is reduced to cysteine to synthesize the antioxidant GSH. Extracellular high concentrations of glutamate and some compounds, such as erastin, analogues, and sorafenib, can be used as inhibitors of System Xc^−^ to consume intracellular cysteine, reduce GSH concentration, cause oxidative stress, increase ROS production, and lead to ferroptosis. Regulating the stability or activity of the System Xc^−^ subunit may be an effective way to regulate cell ferroptosis in the future. Liu et al. found that the ubiquitin hydrolase OTUB1 could control the stability of SLC7A11 and thus regulate cell ferroptosis [33]. In vascular smooth muscle cells (VSMCs), the expression of SLC3A2 helps to stabilize plaque formation and reduce the risk of atherosclerotic thrombosis [34].

## GPX4

GPX4, one of the peroxidase enzymes of GSH, is an important specific marker of ferroptosis. It can maintain the REDOX homeostasis of cells by catalysing the reduction of lipid peroxides or the conversion of free hydrogen peroxide into water, thus protecting cells from oxidative damage [35]. As a specific substrate of GPX4, RSL3 can bind to GPX4 and inactivate it, induce the accumulation of lipid ROS and lead to ferroptosis. Overexpression of GPX4 decreases the sensitivity of RSL3-induced cell ferroptosis [36]. Zhang et al. found that high glucose-induced ferroptosis and cell damage can be regulated by TRIM46 promotion via GPX4 ubiquitin [37]. Recently, Mao et al. revealed that dihydroorotate dehydrogenase (DHODH) could interact with mitochondrial GPX4 to mediate ferroptosis, opening a novel perspective for the mitochondrial pathway of the ferroptosis defence mechanism [38]. Palmitic acid (PA) can reduce the expression levels of heat shock factor 1 (HSF1) and GPX4 in H9c2 cardiomyocytes in a time-dependent and dose-dependent manner. Overexpression of HSF1 can restore intracellular iron homeostasis by regulating iron metabolism-related genes, promoting GPX4 expression and healing the sensitivity of cardiomyocytes to ferroptosis. However, knocking down GPX4 reversed this effect [39].

## Inducers and inhibitors

**Fig. 2:**
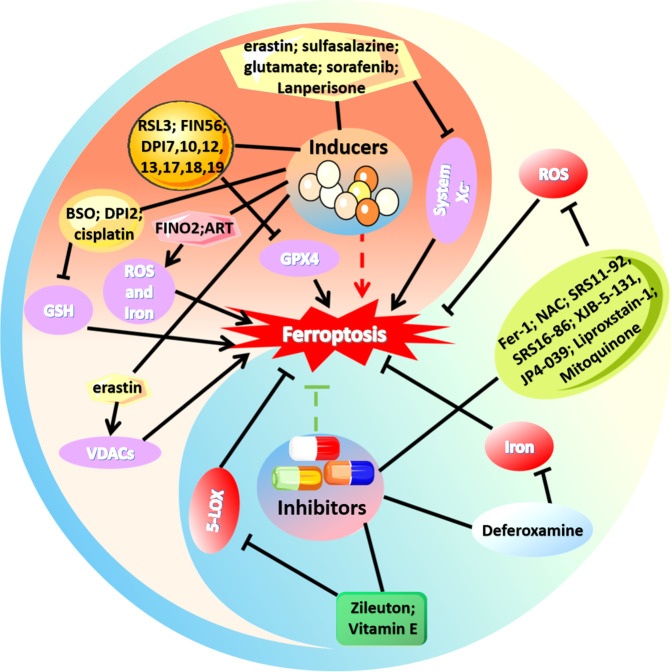
Inducers and inhibitors of ferroptosis. Iron accumulation and ROS production are important signs of ferroptosis. Inhibiting the activity of System Xc^−^ can reduce cystine transport into cells and reduce the synthesis of intracellular GSH, resulting in a decreased ability of GPX4 to scavenge peroxide, increased accumulation of lipid peroxide in cells, and ferroptosis. GSH is an important cofactor of GPX4 activity. GPX4 can scavenge peroxide and maintain the dynamic circulation of GSH in cells, which is a central regulator of ferroptosis; VDAC is located in the outer membrane of mitochondria. Its closure can inhibit the function of mitochondria, change the permeability of the mitochondrial membrane and trigger ferroptosis. LOX-overexpressing cells tend to undergo lipid peroxidation and ROS production and are sensitive to ferroptosis. The arrows indicate promotion, and the blunted lines indicate inhibition.

Ferroptosis is an important form of cell death that is different from other types of cell death in morphology and biochemistry. The mechanism of ferroptosis involves many key factors and signalling pathways. Regulating the decomposition and synthesis of some key molecules can change the sensitivity of cells to ferroptosis. Reasonable induction or inhibition of cell ferroptosis is helpful in improving and treating tumours and CVDs. Several drugs or compounds have been found to induce or inhibit ferroptosis (Fig. 2). According to different targets, ferroptosis inducers can be divided into the following categories: (1) Targeting iron ions and ROS (iron ion oxidation, inactivation of GPX4, induction of ferritin autophagy); (2) targeting System Xc^−^ (inhibit System Xc^−^ activity and prevent GSH synthesis); (3) targeting GSH (reduce glutathione synthesis and inactivate GPX4 by binding with GSH); (4) targeting GPX4 (degrade GPX4 and inhibit GPX4 activity); and (5) targeting voltage-dependent anion channels (VDACs) (reduce GPX4). Inhibitors of ferroptosis can be divided into: (1) targeting iron ions (chelating excess iron); (2) targeting ROS (preventing lipid peroxidation from producing ROS, removing intracellular ROS and inhibiting mitochondrial superoxide generation); and (3) targeting LOX (maintaining cell redox homeostasis). However, the targets and potential applications of these inducers or inhibitors still need to be further studied. In addition, for some compounds with multiple targets, further elucidating their mechanisms, exploring the feasibility of drug combination, and developing more specific inducers or inhibitors will provide better application prospects for clinical treatment.

## Ferroptosis with CVDs

The pathological mechanism of CVDs is complex, and many cell death types are involved. In recent years, ferroptosis has been proven to play an important role in CVDs in continuous studies [40]. Researchers usually assess the impact of ferroptosis in related CVDs by regulating key factors associated with ferroptosis and intervening in the sensitivity of cells to ferroptosis. Here, we summarize the association between various CVDs and ferroptosis (Table 2), such as MI, reperfusion injury, AS, hypertension, myocardial hypertrophy, HF, DCM and DIC.

**Table 2:** The role of ferroptosis in CVDs.

Disease	Factors	Mechanism	Role	Reference
MI	miR-23a-3p	Inhibit DMT1 expression	Inhibit ferroptosis and reduce myocardial injury	[47]
	BACH1	Adjust the threshold of iron ion induction	Inhibit ferroptosis	[48]
Reperfusion injury	C3G	Decreased Fe^2+^, downregulated TfR1 expression, upregulated Fth1 and GPX4 expression	Inhibit ferroptosis and reduce myocardial injury	[57]
	Res	Decreased Fe^2+^, downregulated TfR1 expression, upregulated Fth1 and GPX4 expression	Inhibit ferroptosis and reduce myocardial injury	[58]
	Lip-1	Reduce VDAC1 level and increase GPX4 level	Reduce I/R injury	[126]
	Eto	Induced Nrf2 nuclear translocation	Inhibit I/R-induced ferroptosis, improve fibrosis	[127]
AS	SIRT1	Reduce IL-1β and IL-18 levels	Inhibit ferroptosis and limit AS development	[64]
	PDSS2	Activate Nrf2, inhibit ROS release and reduce iron levels	Promote the proliferation of HCAECs and limit AS development	[65]
	miR-17-92	Targeting zinc lipoprotein A20 reduces Acsl4 expression and ROS accumulation	Inhibit ferroptosis	[66]
	CD98hc	Unknown	Promote VSMCs proliferation and prevent atherosclerotic thrombosis	[34]
Hypertension	Monocrotaline (MCT)	Activate the the HMGB1/TLR4/NLRP3 inflammatory pathway	Promote ferroptosis	[73]
	Celastrol	Increase HO-1 expression and decrease ROS production	Reduce inflammation and oxidative stress in VSMCs caused by hypertension	[74]
	CA	Regulate iron metabolism	Improve PAH	[76]
	Elabela	Regulate the IL-6/STAT3/GPX4 signalling pathway	Inhibit AngII-induced ferroptosis in poor myocardial remodelling, fibrosis and cardiac dysfunction	[79]
Myocardial hypertrophy	DHA	Increase IRF3-SLC7A11, decrease ALOX12 and iron levels	Inhibit ferroptosis	[128]
	miR-351	Regulate the JNK/p53 signalling pathway	Inhibit ferroptosis and improve fibrosis	[84]
	LncRNA AAB	Sponge miR-30b-5p, induced imbalance of MMP9/TIMP1 and enhanced TfR-1	Promote ferroptosis	[86]
HF	CD147	Activate TRAF2-TAK1 signalling pathway	Promote cardiac remodelling and dysfunction	[91]
	TLR4 and NOX4	Unknown	Inhibit cardiac autophagy and ferroptosis in HF rats	[129]
DCM	TRIM46	Promote GPX4 ubiquitination	Resist cell damage caused by high glucose	[37]
	PA	Reduce HSF1 and GPX4	Promote ferroptosis and enhance endoplasmic reticulum stress	[39]
DIC	EMPA	Participate in NLRP3- and MYD88-related pathways	Inhibit ferroptosis, fibrosis, apoptosis and inflammation	[99]
	AsIV	Activate Nrf2 signalling pathway and increase GPX4 expression	Inhibit ferroptosis and improve fibrosis	[100]
Sepsis	LPS	Activate NCOA4 and SFXN1, increase free iron	Cause mitochondrial damage and promote ferroptosis	[108]
	Dexmedetomidine	Reduce HO-1 expression, increase GPX4 expression	Reduce sepsis-induced myocardial cell damage	[107]
Stroke	NCOA4	USP14 upregulates NCOA4 through deubiquitination	Silencing NCOA4 can eliminate the ferritinophagy induced by I/R injury	[102]
	CDKN1A	C9orf106/C9orf139-miR-22-3p-CDKN1A axes	Regulate ferroptosis during IS progression	[104]
	JUN	GAS5-miR-139-5p/miR-429-JUN axes		
	HIF-1α	Inhibits ACSL4 expression in early IS	Against ferroptosis	[105]

## MI

MI refers to injury caused by acute and/or continuous ischaemia and hypoxia of the coronary artery. At present, MI has gradually become one of the main causes of death in patients with CVDs worldwide. Previous reports hold that the adverse consequences of MI mainly include cardiomyocyte apoptosis, necrosis and autophagy [41–43]. However, recent studies have found that the expression of GPX4 is significantly decreased in the early and middle stages of MI [44], suggesting that MI may lead to ferroptosis in myocardial cells. Meanwhile, the downregulation of GPX4 during MI contributes to the ferroptosis of cardiomyocytes under metabolic stress, such as cysteine deprivation [45]. Zhao et al. found that human umbilical cord-derived mesenchymal stem cell exosomes could alleviate acute myocardial infarction (AMI) injury [46]. Song et al. found that the expression of DMT1 was significantly increased in mouse models of AMI and hypoxia-injured myocardial cells [47]. Human umbilical cord blood-derived mesenchymal stem cell exosomes may inhibit DMT1 expression by targeting miR-23a-3p, thereby inhibiting ferroptosis and alleviating MI [47]. The transcription factors BTB and CNC homology 1 (BACH1) are thought to promote ferroptosis at the transcriptional level, and BACH1^−/−^ mice are more resistant to MI than wild-type mice [48]. In addition, ferroptosis often triggers inflammation and leads to the aggravation of cardiac dysfunction and poor myocardial remodelling after MI [49]. Therefore, inhibiting ferroptosis of cardiomyocytes may be a novel avenue for the treatment of MI to improve cardiac function.

## Reperfusion injury

As an important risk factor for CVDs, ischaemia/reperfusion (I/R) injury seriously threatens human life and health [49]. Percutaneous coronary intervention is usually used for MI patients to restore blood flow [50]. Unfortunately, reperfusion may cause further damage to the patients’ heart [51]. Intracellular acidification, anaerobic glucose metabolism and ROS accumulation are involved in the pathological process of I/R damage, and this series of oxidative stress reactions further catalyses the process of lipid peroxidation [52]. Tang et al. hold that ferroptosis occurs mainly in the phase of myocardial reperfusion but not ischaemia [53]. Iron overload in coronary artery flow after I/R leads to the attenuation of cardiac function and the aggravation of myocardial oxidative injury [54]. In the simulated I/R model established by Euncheol et al., ferrostatin-1 (Fer-1) significantly reduced cell death, suggesting that reperfusion injury may cause ferroptosis, which is associated with I/R-induced cell death in vivo [55]. Cyanidin-3-glucoside (C3G), a member of the anthocyanin family, is widely distributed in purple or red vegetables and fruits and has anti-inflammatory, antioxidant, and heart-protecting effects [56]. Shan et al. found that C3G could inhibit ferroptosis in cardiomyocytes by decreasing Fe^2+^ content, downregulating TfR1 expression, and upregulating Fth1 and GPX4 expression, ultimately playing a role in preserving cardiac function [57]. Interestingly, Li et al. found that resveratrol (Res) has a similar function [58]. I/R injury produces oxidized phosphatidylcholines (OxPCs), a bioactive phospholipid intermediate that disrupts mitochondrial bioenergy and calcium transients and triggers cell death through iron overload. Fer-1 or E06 can neutralize OxPCs and prevent cell death during reperfusion [59].

## AS

AS is a chronic progressive and inflammatory artery disease with an intricate pathogenesis, in which dyslipidaemia is the main risk factor, and oxidative stress is a key initiating factor [60]. Endothelial cell dysfunction or death is affected by intracellular lipid peroxides and participates in the regulation of AS [61]. Usually, oxidized low-density lipoprotein (ox-LDL) can induce AS in vitro [62]. Macrophages phagocytose a large number of ox-LDL through surface scavenger receptors, forming foam cells, which are the early lesions of AS [63]. SIRT1 can inhibit ferroptosis of foam cells caused by iron overload through autophagy while reducing the levels of IL-1β and IL-18 and limiting the development of AS [64]. By activating the antioxidant Nrf2, propylene diphosphate synthase subunit 2 (PDSS2) can limit ROS release and iron content to inhibit ferroptosis. Meanwhile, it promotes the proliferation of human coronary artery endothelial cells (HCAECs) and ultimately inhibits the progression of AS [65]. High levels of miR-17-92 in human umbilical vein endothelial cells (HUVECs) can inhibit erastin-induced ferroptosis by targeting zinc lipoprotein A20 to reduce the expression of Acsl4 and the accumulation of ROS [66]. Fer-1 can inhibit the excessive accumulation of iron, alleviate lipid peroxidation and increase the activity of mouse aortic endothelial cells (MAECs) by upregulating the levels of SLC7A11 and GPX4 [67].

## Hypertension

Hypertension is characterized by increased blood pressure in systemic circulation arteries, accompanied by abnormal functions of the heart, brain and kidney [68, 69]. Hypertension is one of the most common chronic diseases and the main risk factor for CVDs [70]. Inflammation triggers vascular remodelling, pulmonary vascular remodelling and increased pulmonary vascular resistance, leading to pulmonary hypertension (PH) [71, 72]. The HMGB1/TLR4 signalling pathway can activate inflammatory bodies in NLRP3 in PH rats, leading to inflammatory ferroptosis, which can be rescued by Fer-1 [73]. Celastrol alleviates cellular inflammation and oxidative stress caused by hypertension through HO-1 induction [74]. Cinnamaldehyde (CA) can regulate vasodilation and resist hypertension caused by insulin deficiency [75]. Zou et al. speculated that celastrol and CA may contribute to the treatment of idiopathic pulmonary arterial hypertension (IPAH) by targeting the iron metabolic pathway [76]. Hydrostatic pressure is one of the main biomechanical forces of blood vessels and plays a key role in the occurrence and development of hypertension [77]. High HP can downregulate Cythionine γ-lyase/H2S in VSMCs; trigger a decrease in GSH levels; and increase iron accumulation, ROS production and lipid peroxidation, which results in aggravation of VSMC dysfunction caused by ferroptosis [78]. Elabela antagonizes cardiac microvascular endothelial cell (CMVEC) ferroptosis by regulating the IL-6/STAT3/GPX4 signalling pathway and improves adverse myocardial remodelling fibrosis and cardiac dysfunction in hypertensive mice [79]. Zhang et al. suggested that the signalling network between miRNAs and transcription factors may be involved in regulating PAH-related ferroptosis, providing a new view to treat hypertension in the future [80].

## Myocardial hypertrophy and HF

HF is one of the leading causes of death worldwide, and its prevalence continues to grow [81, 82]. Adverse cardiac remodelling characterized by pathological myocardial hypertrophy and myocardial fibrosis caused by various extracellular stimuli will eventually develop into HF [83]. The prevention and treatment of pathological myocardial hypertrophy are effective means for the treatment of HF. Mixed lineage kinase 3 (MLK3), a member of the MAP3K family, induces pyroptosis by regulating inflammatory responses mediated by the NF-κB/NLRP3 signalling pathway. Oxidative stress mediated by the JNK/p53 signalling pathway leads to ferroptosis. Pyroptosis and ferroptosis induced by MLK3 lead to the aggravation of myocardial hypertrophy and myocardial fibrosis, contributing to the progression of chronic heart failure (CHF) [84]. Angiotensin II (Ang II) is an important component of the renin angiotensin aldosterone system (RAAS), which can induce cardiomyocyte hypertrophy and reduce xCT expression. Inhibition of xCT exacerbates AngII-induced cardiac hypertrophy and increases the levels of Ptgs2, a biomarker of ferroptosis, malondialdehyde and ROS [85]. LncRNA AAB is upregulated in Ang II-treated CMVECs, which induces MMP9/TIMP1 imbalance, enhances TfR-1 expression and promotes ferroptosis by sponging miR-30B-5p [86]. Patients with HF usually have symptoms of iron deficiency. Systemic iron deficiency leads to reduced iron content in the myocardium, causing insufficient oxygen delivery and erythropoietin resistance, which is one of the risk factors for poor prognosis of patients [87]. Treatment with iron supplements can improve symptoms to some extent, but the exact mechanism is not clear, and it may have potential risks [88]. In Fth-deficient cardiomyocytes, Slc7a11 expression and iron levels were decreased, oxidative stress was increased, and the heart showed mild ageing damage. Because of the low expression of Slc7a11, mice have insufficient ability to regulate iron homeostasis, and a high-iron diet leads to significant ferroptosis characteristics and causes severe heart injury and hypertrophic cardiomyopathy [89]. CD147 is a transmembrane glycoprotein receptor that activates matrix metalloproteinases and promotes inflammation [90]. Zhong et al. demonstrated that CD147 promotes pathological cardiac remodelling and dysfunction in a glycation-dependent manner by binding to the adapter protein TRAF2 and activating the downstream TAK1 signalling pathway, which is accompanied by increased oxidative stress and ferroptosis [91].

## DCM

Diabetes affects cardiac structure and function through a variety of mechanisms, such as metabolic disorders, calcium transport defects, microangiopathy, interstitial fibrosis and cardiac autonomic neuropathy [92]. Oxidative stress is the main cause of DCM, and myocardial hypertrophy and fibrosis are some of its main clinical features [93]. Nrf2 is the main regulator of the cellular REDOX state and detoxification. It can maintain metabolic and redox homeostasis by controlling the expression of specific genomes involved in iron and lipid metabolism and redox balance in the heart [24, 94]. Type 1 diabetes (T1D) causes abnormal cardiac autophagy and interrupts the balance of metabolism and REDOX controlled by Nrf2, inducing ferroptosis of cardiomyocytes, which further aggravates the progression of DCM [94]. At the same time, diabetic patients will produce NOX2-related oxidative stress in an AMPK-dependent way, resulting in ferroptosis and aggravating myocardial I/R injury [95]. Li et al. found that inhibiting endoplasmic reticulum oxidative stress can reduce ferroptosis and cell damage. I/R damage in diabetic mice can be reduced by blocking ferroptosis, which provides a novel therapeutic strategy for myocardial injury [96].

## DIC

DIC is a life-threatening progressive cardiomyopathy caused by DOX cardiotoxicity [97]. DOX is a chemotherapeutic drug for patients with malignant tumours. Its cardiotoxicity can cause ferroptosis and mitochondrial dysfunction [98]. Vincenzo et al. found that EMPA inhibited DOX-induced ferroptosis, myocardial fibrosis and inflammation by participating in NLRP3 and MyD88-related pathways and significantly improved cardiac function in mice [99]. Luo et al. found that astragaloside IV (AsIV) significantly improved DOX-induced myocardial fibrosis and cardiac dysfunction in rats, which may play a role by activating Nrf2 signalling and increasing GPX4 expression [100]. DOX reduces GPX4 expression and induces excessive lipid peroxidation in mitochondria through the DOX-Fe^2+^ complex, resulting in mitochondrial-dependent ferroptosis [98]. Fang et al. confirmed that DOX upregulates Hmox1 through Nrf2-mediated regulation and rapid systematic accumulation of nonheme iron-induced DIC in mice [25]. Targeting mitochondrial oxidative damage may be an effective protective strategy to rescue cardiomyocyte ferroptosis in patients with DIC in the future.

## Stroke

Stroke is an acute cerebrovascular disease that causes brain tissue damage due to insufficient blood supply following cerebral vascular occlusion [101]. Stroke usually causes hemiplegia and disturbance of consciousness, with high mortality and disability rates. It is a global problem that seriously threatens human health. In a model of neuronal I/R injury, ubiquitin-specific peptidase 14 (USP14) increased the expression of NCOA4 in the cytoplasm through deubiquitination. Silencing NCOA4 can eliminate the ferritinophagy induced by I/R injury, thus inhibiting ferroptosis [102]. In brain microvascular endothelial cells (BMVECs) of diabetic animals, iron can increase ROS levels and ferroptosis, which can be prevented by iron chelated deoxyferriamine (DFX) [103]. Fan et al. found that CDKN1A/JUN may be a promising biomarker for the diagnosis of IS and regulate ferroptosis during IS progression through the C9orf106/C9orf139-miR-22-3p-CDKN1A and GAS5-miR-139-5p/miR-429-JUN axes [104]. At the same time, plasma cells may play an important role in the immune microenvironment of IS, which provides a novel approach for the study of therapeutic targets of IS [104]. Yu et al. found that HIF-1α inhibits ACSL4 expression in early IS, which may be derived from the body’s rescue measures against ferroptosis [105]. These results suggest that understanding the role of ferroptosis in IS is of great significance for the prevention and treatment of this devastating disease.

## Others

Studies have shown that sepsis is caused by infection, which can cause cardiac dysfunction, leading to significant morbidity and mortality, and ferroptosis plays an important role in this pathological process [106]. Wang et al. found that the expression of GPX4 decreased and the concentration of iron increased in the hearts of septic mice induced by caecal ligation and puncture (CLP) [107]. In a mouse model of sepsis induced by high-dose lipopolysaccharide (LPS), NCOA4 is activated and releases a large amount of free iron by degrading ferritin. Excessive intracellular Fe^2+^ activates the mitochondrial membrane protein SFXN1, which transports cytoplasmic Fe^2+^ in mitochondria, resulting in the production of mitochondrial ROS and ferroptosis [108]. Abdominal aortic aneurysm (AAA) is a life-threatening vascular disease with a fatality rate of up to 80% [109]. Oxidative stress and inflammation caused by iron overload have been confirmed to contribute to the progression of AAA [110]. In addition, the accumulation of oxidized phospholipids (or their decomposition products) in myocardial tissue of COVID-19 patients also shows the important role of ferroptosis in the progression of heart injury [111].

## Clinical application in CVDs

Ferroptosis is closely related to the occurrence and development of CVDs. Clinical studies have increasingly shown that targeting ferroptosis may be an effective treatment for CVDs. At present, the anti-inflammatory and antioxidant effects of drugs are mainly used to inhibit ferroptosis in the clinic. Deferiprone is an FDA-approved oral active iron chelator that clears intracardial bleeding to relieve hypertrophic heart disease and has cardioprotective effects in acute myocardial infarction [112]. Jiang et al. found that the combination of L-glutamine and deferoxamine can protect the heart from I/R injury [113]. Dexrazoxane is a cyclic derivative of EDTA that easily penetrates the cell membrane and forms a ring-opening iron chelator. As the only drug approved by the FDA to prevent the toxicity of low cumulative dose DOX, it has been proven to successfully inhibit ferroptosis and protect the heart [25, 114]. N-acetylcysteine (NAC) is the donor of glutathione, and the level of GSH decreases under a high oxidation state. NAC can be used to enhance antioxidant therapy [115]. HO-1 can degrade haem into ferrous iron, and overexpression of HO-1 can alleviate hypertrophy, fibrosis and oxidative stress in a failing heart and promote neovascularization [116]. The mechanistic target of rapamycin (mTOR) can protect cardiomyocytes from iron excess and ferroptosis. mTOR is a serine/threonine protein kinase that acts on a large number of iron transporters and participates in the control of iron metabolism [117]. Statins inhibit the biosynthesis of GPX4 and coenzyme Q10, thereby promoting the ferroptosis of mesenchymal cells. Therefore, statins have also been used as an indirect treatment for cardiovascular disease-related ferroptosis [118, 119]. Some natural products with antioxidant activity, such as vitamins, can effectively inhibit ferroptosis and have been shown to have cardioprotective effects [120]. Vitamin E can effectively prevent atherosclerosis, and its potential mechanism may be to prevent ferroptosis by reducing the oxidation of LDL [121]. Puerarin can inhibit lipid peroxidation and iron overload in H9c2 cells, and baicalein can inhibit erastin-mediated GPX4 degradation and enhance the ability of H9c2 cells to resist ferroptosis [122].

## Ferroptosis detection and identification

The morphology of cells can be directly observed by transmission electron microscopy to identify the occurrence of ferroptosis. Iron is an important basic element in the human body and is involved in the maintenance of various physiological functions. During ferroptosis, iron overload occurs in cells, and the detection of significantly increased iron levels can be used as an important indicator to monitor ferroptosis. Recently, an electronic sensing probe compatible with living cells was demonstrated to monitor the dynamics of iron metabolism in real time, which may help us better detect the dynamics of ferroptosis [123]. Iron can produce lipid ROS through the Fenton reaction, causing lipid peroxidation and promoting cell death. Therefore, detecting lipid peroxidation products (such as MDA, LOP, TBARS), ROS levels and cell activity can help us determine ferroptosis. Quantitative polymerase chain reaction (qPCR) or western blot (WB) was used to detect changes in some key factors related to ferroptosis, which can also be used as an important biomarker for determining ferroptosis in cells. In addition, the occurrence of ferroptosis may serve as a biomarker of CVDs and provide important information for the prevention and diagnosis of the disease. The three most important characteristics of biomarkers are specificity, sensitivity and stability. Some indicators of ferroptosis meet these requirements. GPX4 and ACSl4 are two recognized ferroptosis biomarkers [124, 125]. As proteins, ACSl4 and GPX4 are relatively stable in serum; compared with other types of biomarkers, they have the advantage of simple and sensitive determination.

## Discussion

CVDs threaten human health and quality of life. Understanding how cardiomyocyte injury participates in the pathological process of heart-related diseases is the key to formulating heart protection strategies. In recent years, the pathogenic role of iron overload in cardiotoxicity has been widely recognized. Compared with the previously discovered types of cell death, such as apoptosis, necrosis, autophagy and pyroptosis, ferroptosis is an iron-dependent programmed cell death with two obvious biochemical characteristics: intracellular iron accumulation and lipid peroxidation.

Iron metabolism, lipid peroxidation, System Xc^−^ and GPX4 play important roles in the regulation of ferroptosis-related pathways. Abnormal iron metabolism is the main cause of intracellular iron overload, and lipid peroxidation is an important marker of ferroptosis. GPX4 is another important marker of ferroptosis and a key central molecule in System Xc^−^, constituting the metabolic pathway of ferroptosis. As a new type of programmed cell death, the study of ferroptosis involves nervous system diseases, kidney-related diseases, tumours and cardiovascular diseases. In this review, we summarized the regulatory mechanisms of ferroptosis and discussed the role of ferroptosis in CVDs, including MI, reperfusion injury, AS, hypertension, myocardial hypertrophy, HF, DCM and DIC.

Several compounds ameliorate ferroptosis in cardiomyocytes and cardiac dysfunction in CVDs by inhibiting iron accumulation, regulating oxidative stress, and inhibiting lipid peroxidation. However, the specific microscopic reaction targets of these ferroptosis inhibitors are not clear, and whether they have potential toxicity to other organs remains to be confirmed, limiting their clinical application in the treatment of CVDs. At present, studies on ferroptosis are mostly based on animal models and cell levels, and there is still a lack of experimental verification in vivo. Ferroptosis is usually accompanied by an imbalance in ROS signals or an abnormal increase in ROS, which affects cell metabolism and inflammatory signal transduction. However, the specific molecular mechanism by which ROS cause ferroptosis has not been explained in detail. ROS levels, iron concentration, cell viability and some related marker proteins were used to evaluate ferroptosis in experiments. There is still a gap in the accurate detection of ferroptosis progression in vivo. If a specific probe or ferroptosis-related kit can be designed, it will better help in the prevention and treatment of CVDs. In addition, more molecular mechanisms related to ferroptosis remain to be discovered. Gu et al. found that p53 participates in the nonclassical pathway of ferroptosis regulation, which adds more complexity to the research on its mechanism.

In conclusion, ferroptosis is involved in the pathophysiological process of CVDs, suggesting that it can be a potential new drug therapy target. However, further efforts are still required to realize its practical application.
